# Lateral and Basal Amygdala Account for Opposite Behavioral Responses during the Long-Term Expression of Fearful Memories

**DOI:** 10.1038/s41598-017-19074-3

**Published:** 2018-01-11

**Authors:** Eugenio Manassero, Annamaria Renna, Luisella Milano, Benedetto Sacchetti

**Affiliations:** 1 Rita Levi-Montalcini Department of Neuroscience, University of Turin, Corso Raffaello 30, I-10125 Turin, Italy; 2 National Institute of Neuroscience, Turin, Italy

## Abstract

Memories of fearful events can be maintained throughout the lifetime of animals. Here we showed that lesions of the lateral nucleus (LA) performed shortly after training impaired the retention of long-term memories, assessed by the concomitant measurement of two dissociable defensive responses, freezing and avoidance in rats. Strikingly, when LA lesions were performed four weeks after training, rats did not show freezing to a learned threat stimulus, but they were able to direct their responses away from it. Similar results were found when the central nucleus (CeA) was lesioned four weeks after training, whereas lesions of the basal nucleus (BA) suppressed avoidance without affecting freezing. LA and BA receive parallel inputs from the auditory cortex, and optogenetic inhibition of these terminals hampered both freezing and avoidance. We therefore propose that, at variance with the traditional serial flow of information model, long-term fearful memories recruit two parallel circuits in the amygdala, one relying on the LA-to-CeA pathway and the other relying solely on BA, which operate independently and mediate distinct defensive responses.

## Introduction

The association between a neutral sensory stimulus (the conditioned stimulus, CS, such as a tone) and a painful event (the unconditioned stimulus, US, for example a mild footshock) is rapidly formed and can be maintained throughout the lifetime of animals. The neural basis for the encoding of CS-US association is well studied. Information about the CS and US is thought to converge in the lateral nucleus of the amygdala (LA), where neurons undergo learning-evoked changes. This information is then conveyed to the basal nucleus (BA) and the central nucleus (CeA), which coordinate the expression of defensive behaviors^1–4^. In particular, CeA outputs are crucial for the expression of passive autonomic responses to the CS (including freezing) while it is dispensable for the avoidance of threat stimuli^5–8^. Conversely, BA is not necessary for the expression of freezing^5,9,10^ but see^11^ while it plays a crucial role in the active responses, like “escape from fear” and avoidance^5–8,12^.

This model of serial information flow within the amygdala is based on anatomical tracing, lesion, pharmacological, electrophysiological and optogenetic studies performed during or shortly after CS-US association. On the other hand, it is less clear whether this serial flow of information processing is maintained in the course of consolidation and long-term maintenance of fearful memories. Several studies showed that the neural circuits underlying fearful memory maintenance gradually reorganize over time^13–16^. Based on evidence indicating that the retrieval of long-term fearful memories enhances LA activity^17,18^ but see^15^ and that combined lesions in both LA and BA impair fearful memories across the lifetime of animals^19–21^, it has been proposed that CS-US association is permanently stored in LA^22^. However, there have been no studies addressing the individual involvement of LA or BA nuclei in the long-term expression of fearful memories, and their individual contributions to this process remain poorly understood. In particular, it remains a matter of debate whether the LA persists in being a crucial locus of memory as time passes^22–24^.

Therefore, in the present study, we investigated the involvement of different amygdala nuclei during the formation versus the long-term expression of fearful memories assessed through the simultaneous examination of two dissociable defensive behaviors, freezing and avoidance responses.

## Results

### LA lesions differently affected the formation and the long-term expression of fearful memories

**Figure 1:**
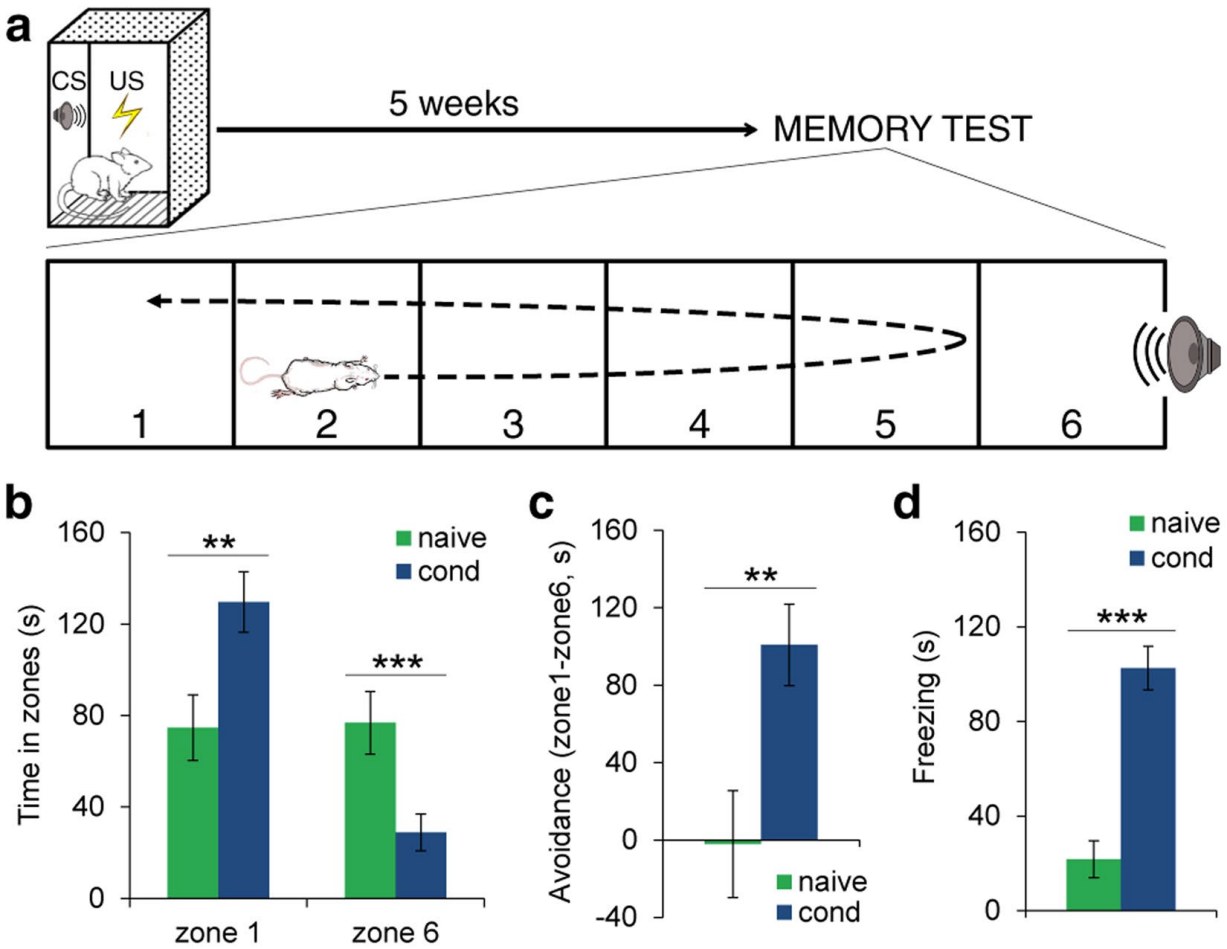
A new procedure to test simultaneously the avoidance behavior and freezing response to threatening conditioned stimuli in the same animals. (**a**) Rats underwent conditioning to associate auditory stimuli (CS) with unconditioned painful stimuli (US). Long-term memories were tested in a cage that allowed the expression of freezing responses and avoidance of the CS. (**b**) Time spent in zone 1 and zone 6 during the memory test in naive (*n* = 13) and conditioned (*n* = 15) rats. Conditioned animals spent more time in zone 1 and less time in zone 6 than naive animals. (**c**) Avoidance behavior (seconds spent in zone 1 minus seconds spent in zone 6) was stronger in conditioned versus naive animals. Naive rats spent approximately the same amount of time in the two different zones, showing no avoidance behavior. (**d**) The freezing response to the CS in conditioned rats was greater than in naive animals. *P < 0.05; **P < 0.01; ***P < 0.005. All values are the mean ± SEM.

Because previous studies showed that different amygdala nuclei may be involved in the expression of different defensive responses^5–8,12^, we set-up a new behavioral paradigm which allows the concomitant measurement of two dissociable defensive responses, freezing and avoidance. Rats were trained to associate a tone (CS) with a footshock (US) in a standard conditioning chamber. One month later, the CSs were presented within a new apparatus divided into six subzones (see Methods) (Fig. 1a). The CS was delivered in zone 6. In this situation, the rats directed their actions to actively avoid the CS by running away from zone 6 towards the zone farthest from the CS (zone 1). Compared with naive (*n* = 13) rats, animals that underwent fear conditioning (*n* = 15) spent more time in zone 1 and less time in zone 6 (Fig. 1b). A 2 × 2 mixed ANOVA showed a significant main effect of the zone (*F*_(1,26)_ = 8.31, *P* = 0.008). Student’s *t* tests revealed that naive animals differed from conditioned animals in zone 1 (*t*_(26)_ = −2.82, *P* = 0.009) and in zone 6 (*t*_(26)_ = 3.12, *P* = 0.004). To further characterize the overall level of avoidance, we calculated an avoidance index by subtracting the time spent in zone 6 from the time spent in zone 1 (Fig. 1c). Avoidance behavior was stronger in conditioned versus naive animals (*t*_(26)_ = 3.00, *P* = 0.006). Naive rats spent approximately the same amount of time in the two different zones, showing no avoidance behavior. Upon arrival in zone 1, the conditioned rats began to freeze and the freezing response in conditioned rats was greater than in naive animals (*t*_(26)_ = 6.57, *P* < 0.001) (Fig. 1d). Thus, our paradigm allows the assessment of fearful memory by simultaneously measuring two fear indices in the same animal: the time spent in zone 1 to avoid the CS and the time spent freezing. This procedure differs from those employed in previous studies in which fearful memory retention was assessed in a cage where the animals were only able to freeze, because alternative defensive behaviors, such as aversion or escape, were not viable.

**Figure 2:**
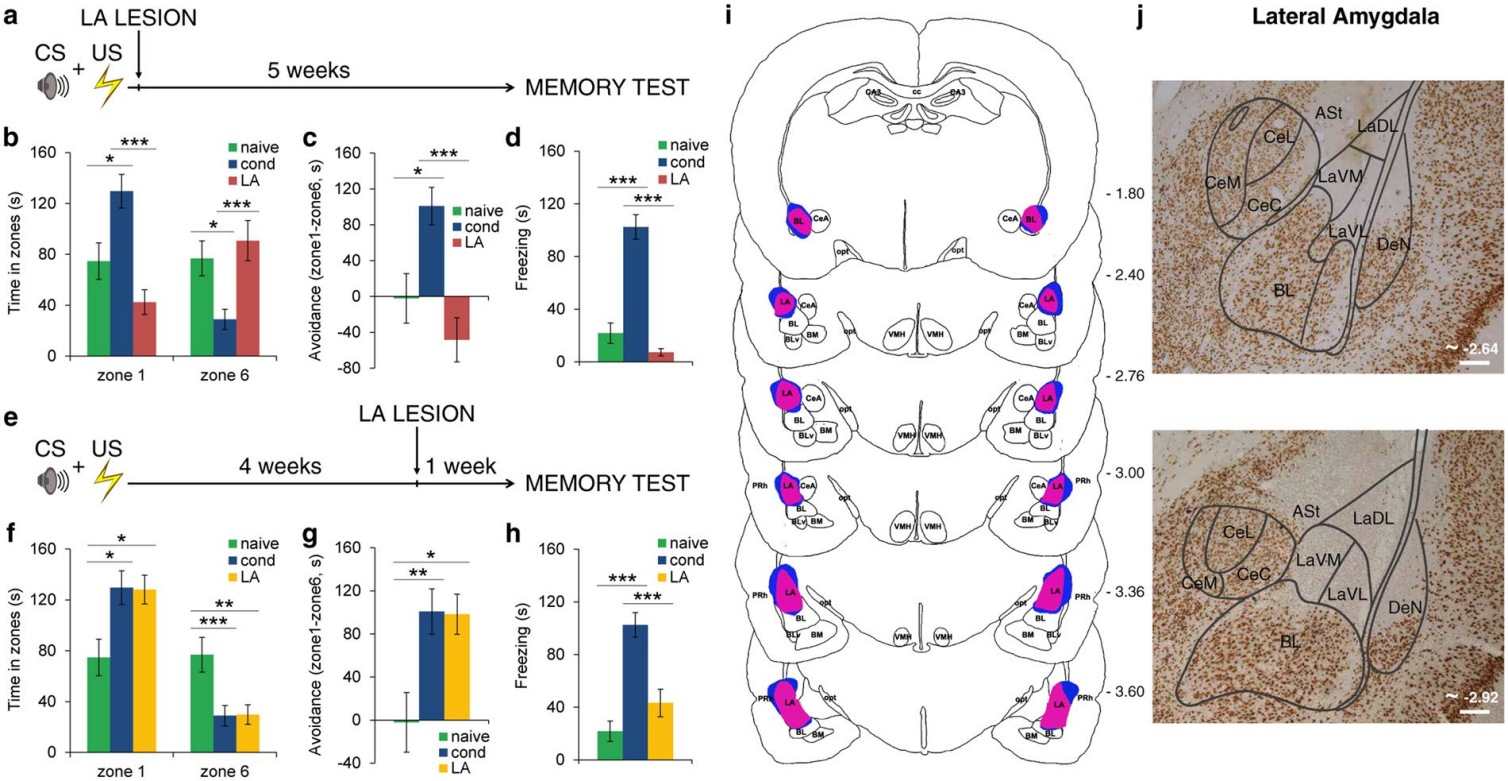
The role of LA in the formation and the long-term expression of fearful memories. (**a**) LA was lesioned 15–20 mins after training. Long-term memories were tested five weeks later. (**b**) The time spent in zone 1 by rats lesioned in the LA 15–20 mins after conditioning (*n* = 11) was lower than conditioned ones, while it was higher in zone 6. (**c**) Contrary to the conditioned rats, animals that were LA-lesioned 15–20 mins after training showed no avoidance of the source of the threat. (**d**) When animals were damaged in the LA 15–20 mins after conditioning, the freezing response was abolished. (**e**) LA was lesioned four weeks after training. Long-term memories were tested one week later. (**f**) LA-lesioned rats (*n* = 12) were able to direct their behavior away from the source of the CS. The time spent in zone 1 by LA-lesioned rats was significantly greater than for naive animals and was reduced in zone 6, but it did not differ from the time spent by conditioned rats in both instances. (**g**) Similarly, conditioned animals and LA-lesioned rats spent more time displaying avoidance behavior. (**h**) On the other hand, the freezing behavior of LA-lesioned rats was low and similar to that of naive animals. (**i**) Representations of the smallest (red-violet) and the widest (blue) excitotoxic damage of LA. Negative numbers indicate posterior distance from bregma. The serial section diagram was drawn on the basis of our NeuN-stained sections. (**j**) Representative photomicrographs of LA lesions. Scale bars, 200 μm. *P < 0.05; **P < 0.01; ***P < 0.005. All values are reported as the mean ± SEM. ASt, amygdalostriatal transition; BL, basolateral amygdaloid nucleus; BLv, basolateral amygdaloid nucleus, ventral part; BM, basomedial amygdaloid nucleus; CA3, field CA3 of the hippocampus; cc, corpus callosum; CeA, central amygdaloid nucleus; CeC, central amygdaloid nucleus, capsular division; CeL, central amygdaloid nucleus, lateral division; CeM, central amygdaloid nucleus, medial division; DeN, dorsal endopiriform nucleus; LA, lateral amygdaloid nucleus; LaDL, lateral amygdaloid nucleus, dorsolateral part; LaVL, lateral amygdaloid nucleus, ventrolateral part; LaVM, lateral amygdaloid nucleus, ventromedial part; opt, optic tract; PRh, perirhinal cortex; VMH, ventromedial hypothalamic nucleus. See also Supplementary Fig. S1.

We thus exploited this paradigm to investigate the involvement of LA in the formation or the long-term expression of fearful memories. To do this, LA was lesioned 15–20 mins after training or four weeks after training. In both conditions, memory was tested one month after CS-US association (Fig. 2a and e). Lesions entirely ablated the dorsal, medial and ventral subregions of LA but spared the BA and CeA nuclei (Fig. 2i,j and Supplementary Fig. S1). In keeping with previous studies^1,5,8^, LA lesions performed 15–20 mins after training (*n* = 11) impaired the formation of long-term memories, regardless of whether or not freezing (Fig. 2d) or avoidance (Fig. 2b and c) was used as a measure of memory retention. A mixed ANOVA model yielded a significant effect of the group in zone 1 (*F*_(2,36)_ = 11.681, *P* < 0.001) and zone 6 (*F*_(2,36) = _7.312, *P* = 0.002). *Post hoc* Bonferroni-corrected comparisons revealed that LA-lesioned animals spent less time in zone 1 (*P* < 0.001) and more time in zone 6 (*P* = 0.003) than conditioned ones, but they did not differ from naive ones (*P* > 0.05) in both zones. LA-lesioned animals showed a lack of avoidance behavior (one-way ANOVA, *F*_(2,36)_ = 10.036, *P* < 0.001; conditioned versus LA-lesioned rats, *P* < 0.001, naive versus LA-lesioned rats, *P* > 0.05) and a freezing response lower than conditioned ones but similar to naive ones (one-way ANOVA, *F*_(2,36)_ = 45.821, *P* < 0.001; conditioned versus LA-lesioned rats, *P* < 0.001, naive versus LA-lesioned rats, *P* > 0.05).

We then repeated similar experiments but by lesioning LA four weeks after training. In line with previous studies^20,21^, upon delivery of the CS, LA-lesioned rats (*n* = 12) did not display conditioned freezing responses (Fig. 2h). The freezing of LA-lesioned rats was low and similar to that of naive animals (one-way ANOVA, *F*_(2,37)_ = 21.9, *P* < 0.001; conditioned versus LA-lesioned rats, *P* < 0.001, naive versus LA-lesioned rats, *P* > 0.05). Strikingly, however, the same animals were still able to bias their actions away from the CS and spent significantly more time in the zone farthest away (Fig. 2f). Mixed ANOVA revealed main effects of the zone (*F*_(1,37) = _24.5, *P* < 0.001) and the group in zone 1 (*F*_(2,37)_ = 5.60, *P* = 0.007) and zone 6 (*F*_(2,37) = _7.29, *P* = 0.002). Pairwise Bonferroni-corrected comparisons indicated that the time spent in zone 1 by LA-lesioned rats was significantly greater than for naive animals (*P* = 0.026) and was reduced in zone 6 (*P* = 0.009) but did not differ from the time spent by conditioned rats (*P* > 0.05 in both instances). These data were further confirmed by the avoidance index (Fig. 2g). Conditioned animals and LA-lesioned rats spent more time displaying avoidance behavior (*F*_(2,37) = _6.52, *P* = 0.004; naive versus LA-lesioned rats, *P* = 0.014; conditioned versus LA-lesioned animals, P > 0.05). These data revealed that during the retrieval of long-term fearful memories the LA is necessary for the expression of freezing, but not the avoidance of threatening CSs.

Thus, LA involvement in the processing of CS-US association changes over the course of the formation and the long-term maintenance of this information. LA is essential for the formation of fearful memories whereas, as time passes, it becomes necessary only for those memories which require the expression of the freezing response.

### Two independent pathways within the amygdala support the expression of freezing and avoidance during the retrieval of long-term memories

**Figure 3:**
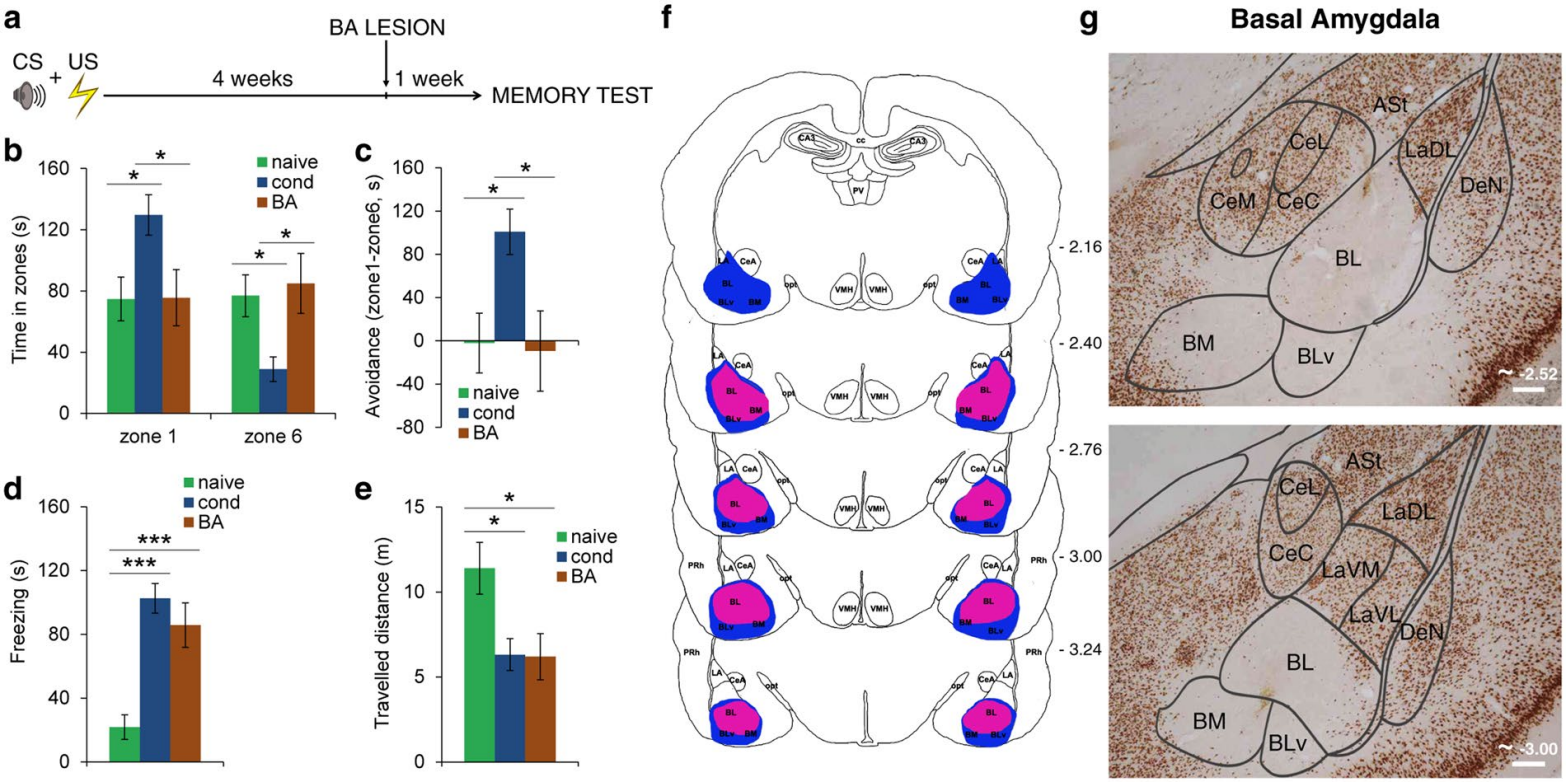
The role of BA in the expression of long-term fearful memories. (**a**) BA was lesioned four weeks after training and long-term memories were tested one week later. (**b**) Time spent in zone 1 and zone 6 by BA-lesioned rats (*n* = 13). The time spent in zones 1 and 6 was similar between naive and BA-lesioned rats, and significantly differed between conditioned and BA-lesioned rats in zone 1 and in zone 6. (**c**) The avoidance index analysis revealed differences between BA-lesioned and conditioned rats, while no differences were found between BA-lesioned and naive rats. (**d**) BA lesions did not affect conditioned freezing responses to the CS. (**e**) The travelled distance in BA-lesioned rats was significantly lower than in naive ones, while it was not different from conditioned animals. (**f**) Extent of the narrowest (red-violet) and the largest (blue) lesion of the basal nucleus of the amygdala (BA). Negative numbers indicate posterior distance from bregma. The serial section diagram was drawn on the basis of our NeuN-stained sections. (**g**) Representative photomicrographs of NeuN-staining of the BA lesion induced by NMDA administration. Scale bars, 200 μm. *P < 0.05; **P < 0.01; ***P < 0.005. All values are reported as the mean ± SEM. ASt, amygdalostriatal transition; BL, basolateral amygdaloid nucleus; BLv, basolateral amygdaloid nucleus, ventral part; BM, basomedial amygdaloid nucleus; CA3, field CA3 of the hippocampus; cc, corpus callosum; CeA, central amygdaloid nucleus; CeC, central amygdaloid nucleus, capsular division; CeL, central amygdaloid nucleus, lateral division; CeM, central amygdaloid nucleus, medial division; DeN, dorsal endopiriform nucleus; LA, lateral amygdaloid nucleus; LaDL, lateral amygdaloid nucleus, dorsolateral part; LaVL, lateral amygdaloid nucleus, ventrolateral part; LaVM, lateral amygdaloid nucleus, ventromedial part; opt, optic tract; PRh, perirhinal cortex; PV, paraventricular thalamic nucleus; VMH, ventromedial hypothalamic nucleus. See also Supplementary Fig. S2.

Our findings raise the question of which neural pathway is recruited to actively avoid learned threat stimuli during the long-term expression of fearful memories. Previous studies showed that during the formation of fearful memories BA is required to learn to escape from fear^5^ and to avoid threats^6–8,12^. Thus, it is possible that BA covers this role also during the expression of long-term memories. However, in this case, it should become independent from information of LA, that our findings showed to not be necessary for the avoidance of CSs. In order to shed light on this issue, we lesioned BA four weeks after training and we tested lesioned rats as in the case of LA-lesioned ones. BA lesions included the basomedial and basolateral regions, and spared LA and CeA nuclei (Fig. 3f,g and Supplementary Fig. S2). In marked contrast to the results observed in LA-lesioned animals, BA-lesioned rats (*n* = 13) did not display any avoidance behavior in response to the CS (Fig. 3b and c). A mixed ANOVA showed a significant main effect of the group in zone 1 (*F*_(2,38)_ = 4.45, *P* = 0.018) and in zone 6 (*F*_(2,38)_ = 4.86, *P* = 0.013). Pairwise comparisons showed that the time spent in zones 1 and 6 was similar between naive and BA-lesioned rats (*P* > 0.05) and significantly differed between conditioned and BA-lesioned rats in zone 1 (*P* = 0.046) and in zone 6 (*P* = 0.021). Furthermore, the avoidance index analysis (*F*_(2,38)_ = 4.82, *P* = 0.014) revealed differences between BA-lesioned and conditioned rats (*P* = 0.028). However, it is noteworthy that although the averaged time spent in zones 1 and 6 was similar between BA-lesioned and naive rats, the behavior of the two groups differed markedly. In fact, upon delivery of the CS, BA-lesioned rats moved to the nearest extremity of the cage, irrespective of the source of the CS, and then remained there. That is, some animals (*n* = 6) moved to zone 1 and then spent most of their time there, whereas others (*n* = 5) moved to zone 6 and remained there. To bring out these differences, we analysed the overall distance travelled by naive and BA-lesioned animals (Fig. 3e). The two groups differed significantly whilst BA-lesioned rats were similar to the conditioned animals (one-way ANOVA, *F*_(2,38)_ = 5.34, *P* = 0.009; BA-lesioned versus naive rats, *P* = 0.022, BA-lesioned versus conditioned groups, *P* > 0.05). The difference between BA-lesioned rats and naive rats became evident when we considered freezing in response to the CS. While naive animals showed very little freezing, BA-lesioned rats showed higher levels of freezing, similar to those in the conditioned animals (one-way ANOVA, *F*_(2,38)_ = 16.2, *P* < 0.001; BA-lesioned versus naive rats, *P* < 0.001, BA-lesioned versus conditioned group, *P* > 0.05) (Fig. 3d). Thus, rats with BA lesions were unable to direct their actions to avoid the CS. Upon delivery of the CS, they suddenly began to freeze. These results showed that at variance with LA lesions, BA lesions impaired the avoidance of the CS, but the conditioned freezing response remained intact. Therefore, several weeks after learning BA has become independent from LA information and the two nuclei mediate different behavioral responses.

**Figure 4:**
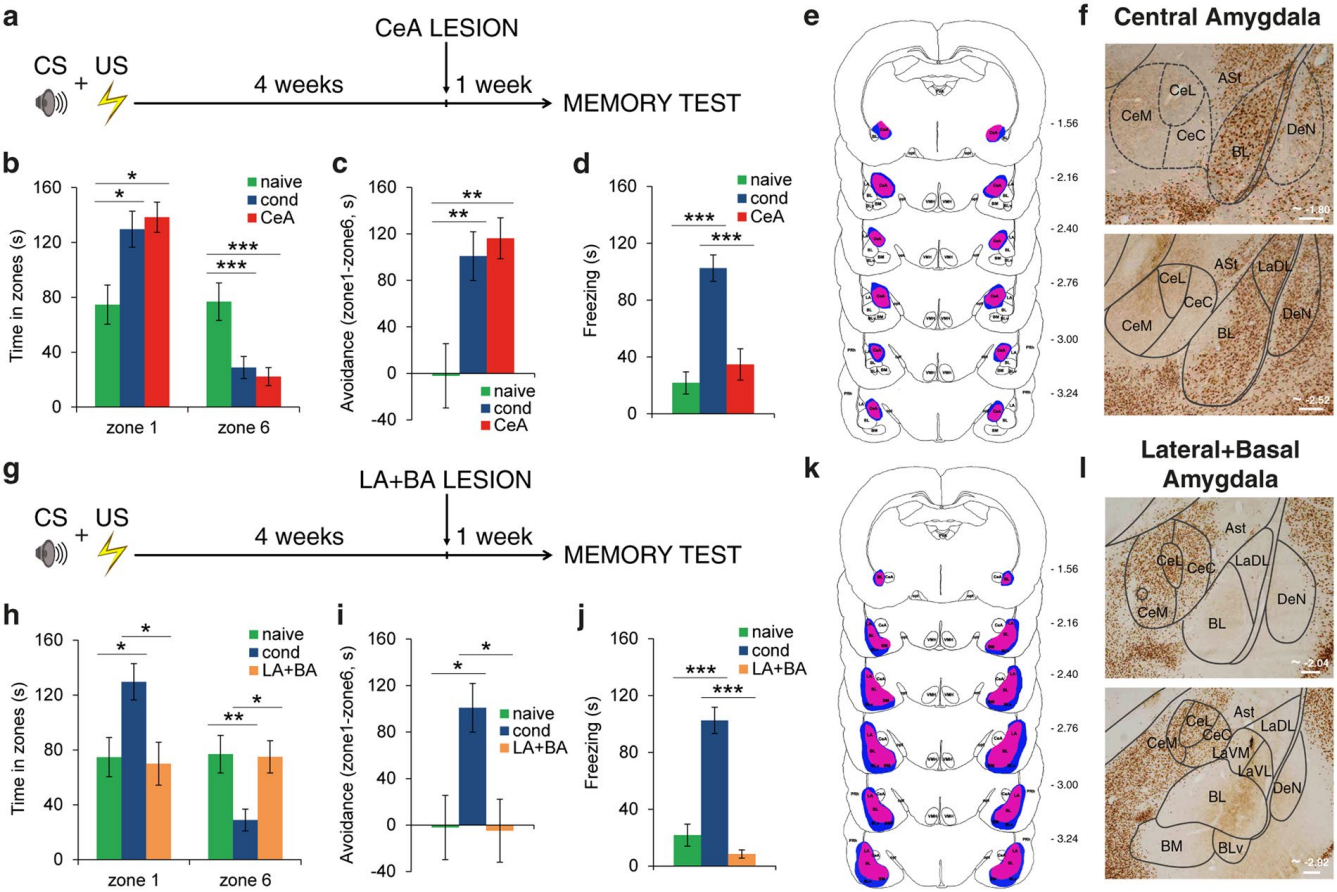
Long-term expression of fearful memories in animals with CeA or LA + BA lesions. (**a**) Rats were lesioned in the CeA four weeks after training. (**b**) Time spent in zone 1 and zone 6 by naive, conditioned and CeA-lesioned rats (*n* = 9). CeA-lesioned animals differed from naive animals in zone 1 and in zone 6, whereas there were no differences between CeA-lesioned and conditioned rats. (**c**) CeA-lesioned rats showed significant CS avoidance, similarly to conditioned animals. (**d**) Freezing behavior in CeA-lesioned rats was lower than in the conditioned group and similar to that in naive animals. (**e**) Reconstructions of the narrowest (red-violet) and the largest (blue) excitotoxic lesion of the CeA. Negative numbers indicate posterior distance from bregma. The serial section diagram was drawn on the basis of our NeuN-stained sections. (**f**) Representative photomicrographs of NeuN-staining of CeA damage. Scale bars, 200 μm. (**g**) Rats received combined lesions of LA + BA four weeks after training. (**h**) Time spent in zone 1 and zone 6 during the memory test, by naive, conditioned and LA + BA-lesioned rats (*n* = 8). A mixed ANOVA revealed a significant main effect of the zone (*F*_(1,33)_ = 4.27, *P* = 0.047), significant main effects of the group in zone 1 (*F*_(2,33)_ = 5.68, *P* = 0.008) and in zone 6 (*F*_(2,33)_ = 6.40, *P* = 0.004). Rats that underwent a lesion in the LA + BA spent less time in zone 1 (*P* = 0.030) and more time in zone 6 (*P* = 0.033) than conditioned subjects, but did not differ from naive rats (*P* > 0.05 in both instances). (**i**) Similarly to naive subjects, avoidance behavior (*F*_(2,33)_ = 6.17, *P* = 0.005) was lower in LA + BA lesioned rats relative to conditioned ones (*P* = 0.028). (**j**) Freezing responses in animals lesioned in the LA + BA (*F*_(2,33)_ = 38.7, *P* < 0.001) were lower than conditioned ones (*P* < 0.001) but similar to naive ones (*P* > 0.05). (**k**) Reconstructions of the extension of LA + BA lesions. Smallest damage is red-violet painted, largest is blue painted. The serial section diagram was drawn on the basis of our NeuN-stained sections. (**l**) Representative photomicrographs of NeuN-staining of the LA + BA lesion. Scale bars, 500 μm. *P < 0.05; **P < 0.01; ***P < 0.005. All values are reported as the mean ± SEM. ASt, amygdalostriatal transition; BL, basolateral amygdaloid nucleus; BLv, basolateral amygdaloid nucleus, ventral part; BM, basomedial amygdaloid nucleus; cc, corpus callosum; CeA, central amygdaloid nucleus; CeC, central amygdaloid nucleus, capsular division; CeL, central amygdaloid nucleus, lateral division; CeM, central amygdaloid nucleus, medial division; DeN, dorsal endopiriform nucleus; LA, lateral amygdaloid nucleus; LaDL, lateral amygdaloid nucleus, dorsolateral part; LaVL, lateral amygdaloid nucleus, ventrolateral part; LaVM, lateral amygdaloid nucleus, ventromedial part; opt, optic tract; PRh, perirhinal cortex; PVA, paraventricular thalamic nucleus, anterior part; VMH, ventromedial hypothalamic nucleus. See also Supplementary Fig. S3.

These data raise the question of the involvement in the expression of long-term memories of the other nucleus of the amygdala that has been studied extensively in fear conditioning, namely the CeA. A recent study showed that this nucleus plays a crucial role in the retrieval of long-term fearful memories tested by analyzing the freezing response^15^. We therefore tested the effects of lesioning the CeA four weeks after training in our behavioral paradigm. Lesions encompassed the lateral, capsular and medial divisions of CeA, but spared LA and BA (Fig. 4e,f and Supplementary Fig. S3). Upon delivery of the CS, CeA-lesioned rats (*n* = 9) were able to bias their actions away from the CS and spent significantly more time in the zone farthest away (Fig. 4b and c). A 3 × 2 mixed ANOVA yielded a significant main effect of the zone (*F*_(1,34) = _26.3, *P* < 0.001), significant effects of the group in zone 1 (*F*_(2,34)_ = 6.38, *P* = 0.004) and in zone 6 (*F*_(2,34) = _8.09, *P* = 0.001). Pairwise Bonferroni-corrected comparisons revealed that CeA-lesioned animals differed from naive animals in zone 1 (*P* = 0.012) and zone 6 (*P* = 0.005), whereas there were no differences between CeA-lesioned and conditioned rats (*P* > 0.05 in both zones). These data were confirmed by the avoidance analysis revealing significant CS avoidance in the CeA-lesioned rats. One-way ANOVA showed significant differences among groups (*F*_(2,34)_ = 7.33, *P* = 0.002) and Bonferroni *post hoc* comparisons indicated differences between CeA-lesioned and naive rats (*P* = 0.007) but not between conditioned and CeA-lesioned rats (*P* > 0.05). At the same time, the conditioned freezing response was abolished in these rats (Fig. 4d). Freezing behavior in CeA-lesioned rats (*F*_(2,34)_ = 24.3, *P* < 0.001) was similar to that in naive animals (*P* > 0.05) and different from that in the conditioned group (*P* < 0.001). This result suggests that CeA is necessary for the expression of freezing, but not avoidance behavior, in marked similarity to LA-lesioned rats and in contrast to BA-lesioned rats.

This double dissociation leads to the idea that during the expression of long-term memories there are two distinct and dissociable neural systems within the amygdala (LA-to-CeA and BA) that are not only independently capable of supporting the retention of memory information, but also independently mediate distinct types of defensive responses. The existence of these parallel circuits implies that the inhibition of the entire amygdala, encompassing the CeA, LA and BA, or even LA (which triggers CeA activity) and BA nuclei alone, should block the expression of both freezing and avoidance responses. Consistent with this assumption, animals with combined lesions of LA and BA (Fig. 4h–l) or of the entire amygdala (Supplementary Fig. S5) were totally impaired in both fear-related responses.

### LA and BA nuclei receive parallel and independent inputs from the auditory cortex during the expression of long-term memories

**Figure 5:**
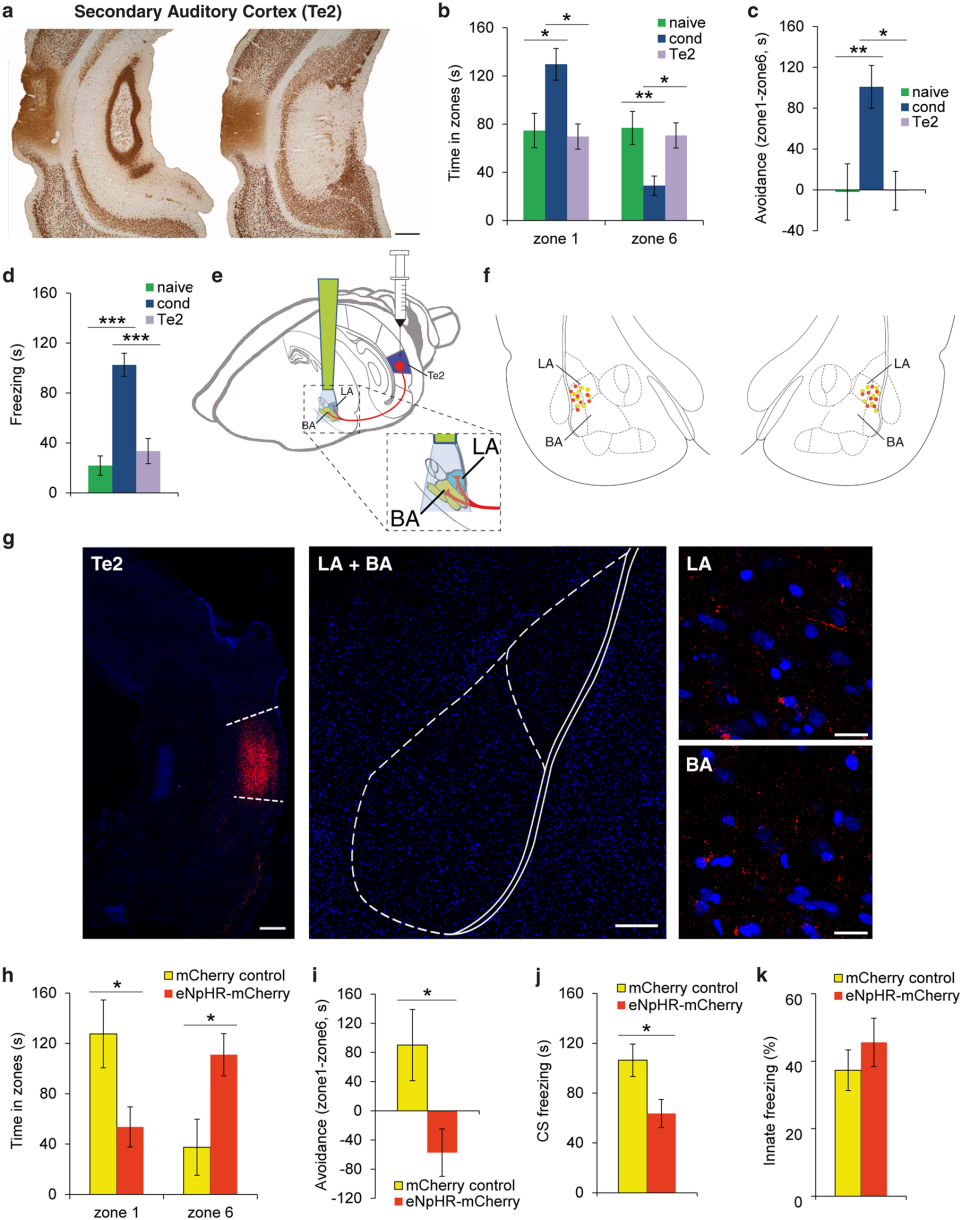
The higher-order Te2 cortex provides essential information to LA and BA during auditory memory retrieval. (**a**) Lesions were centered in the secondary auditory cortex Te2 (*n* = 9). Scale bar, 500 μm. (**b**) A mixed-design ANOVA indicated a significant main effect of the zone (*F*_(1,34) = _5.36, *P* = 0.027), significant main effects of the group in zone 1 (*F*_(2,34)_ = 6.48, *P* = 0.004) and zone 6 (*F*_(2,34) = _6.27, *P* = 0.005). Pairwise comparisons showed significant differences between Te2-lesioned and conditioned animals in zones 1 (*P* = 0.015) and 6 (*P* = 0.044). Indeed, the distribution of the time spent in zones 1 and 6 was similar in Te2-lesioned and naive animals (*P* > 0.05). (**c**) Avoidance behavior was weaker (*F*_(2,34) = _6.68, *P* = 0.004) in Te2-lesioned animals relative to conditioned rats (*P* = 0.020). (**d**) The freezing responses of Te2-lesioned rats were similar to naive animals (*F*_(2,34)_ = 25.6, *P* < 0.001; naive versus Te2-lesioned animals, *P* > 0.05; Te2-lesioned versus conditioned rats, *P* < 0.001). (**e**) The Te2 region of the rats was injected with rAAV5/CamKIIa-eNpHR3.0-mCherry-WPRE. (**f**) Bilateral placement of optic fiber tips within the LA and BA. Orange dots represent eNpHR-mCherry-injected animals, while yellow dots represent mCherry control animals. (**g**) Representative micrographs showing the expression of eNpHR3.0-mCherry (in red) in Te2 and its terminals in either LA and BA. Nuclei were counterstained with DAPI. Scale bars, 300, 200, 20, 20 μm respectively. (**h**) eNpHR-mCherry-injected animals (*n* = 8) spent significantly less time in zone 1 and significantly more time in zone 6 than mCherry controls (*n* = 8). (**i, j**) Animals injected with eNpHR-mCherry exhibited significantly lower (**i**) avoidance and (**j**) freezing than mCherry controls. (**k**) There were no differences in the freezing response to the presentation of an innate threat stimulus. *P < 0.05; **P < 0.01; ***P < 0.005. All values are reported as the mean ± SEM. See also Supplementary Fig. S4.

Our findings raise the question of which inputs carry the mnemonic information about learned stimuli to BA and LA. The amygdala receives inputs from the auditory cortex, especially from the higher-order Te2 and Te3 areas^25–27^. Recent findings suggested that the Te2 cortex is an essential locus for the long-term maintenance of auditory fearful memories^14,28–31^, and that Te2 activity is highly correlated with amygdala activity during long-term memory expression^32^. Intriguingly, the Te2 cortex sends projections not only to LA but also to BA^25–27^. Therefore, this cortex may provide information about auditory fearful memories to both LA and BA. To test this hypothesis, we first investigated the impact of Te2 lesions on fearful memories using our behavioral paradigm. Te2-lesioned rats did not display the avoidance behavior (Fig. 5b and c) and freezing response towards the CS (Fig. 5d), suggesting that this region is essential for the long-term memory expression, irrespective of the fear index measured. However, the data do not demonstrate that Te2 is the source of information for either LA and BA. To address this question directly, we relied on an optogenetic approach to specifically silence Te2 terminals in LA and BA. In Te2, we administered an adeno-associated viral vector (AAV-5) expressing the light-sensitive chloride pump halorhodopsin combined with enhanced red fluorescent protein (mCherry). The virus was under the control of a CaMKIIα promoter (AAV5-CaMKIIa-eNpHR3.0-mCherry) that drives its expression within pyramidal neurons^33^, i.e. the neurons that are the major source of efferents to subcortical nuclei^25–27^. Several weeks after injection of the viral vector, we observed Te2 terminals labeled in both LA and BA (Fig. 5e and g). We then implanted optical fibers and, one week later, we delivered light in order to simultaneously inactivate Te2 terminals in LA and BA during the long-term memory retention test. The inhibition of Te2 terminals in LA and BA impaired both the avoidance of the CS (Fig. 5h and i) and the conditioned freezing response (Fig. 5j). Student’s *t* test showed that eNpHR-mCherry-injected animals (*n* = 8) spent significantly less time in zone 1 (*t*_(14)_ = −2.36, *P* = 0.033) and significantly more time in zone 6 (*t*_(14)_ = 2.64, *P* = 0.019) than mCherry controls (*n* = 8). Accordingly, animals injected with eNpHR-mCherry exhibited significantly lower avoidance than control animals (*t*_(14)_ = −2.50, *P* = 0.025). Furthermore, animals injected with eNpHR-mCherry showed lower levels of freezing than mCherry controls (*t*_(14)_ = −2.48, *P* = 0.026). Thus, these data suggested that Te2 axons carry information about fearful memories that is essential to LA for the expression of freezing responses and to BA for the regulation of avoidance behaviors. Alternatively, optogenetic manipulations may have impaired the basal activity of the amygdala, thereby preventing the expression of defensive responses. To rule out this possibility, we probed the effects of Te2 terminal inhibition during the presentation of an innate threat stimulus, such as the image of a predator, in the same rats. In this situation, the inhibition of Te2 terminals did not affect defensive responses (*t*_(14)_ = 0.88, *P* > 0.05) (Fig. 5k). These data showed that optogenetic inhibition of Te2 terminals in LA and BA impaired the expression of fear-related behaviors towards learned auditory CSs while it did not affect amygdala activity in the presence of innate threats. In addition, these data showed that not only the lesioning procedure but also the optogenetic inactivation of LA + BA affected long-term memories.

## Discussion

Here we showed that LA lesions resulted in different effects depending on the memory phase in which they were performed. LA lesions performed 15–20 mins after training impaired long-term fearful memories regardless of whether or not freezing or avoidance was used as a measure. Conversely, when lesions of LA were performed four weeks after training, only the freezing response, but not the avoidance, was impaired. At this late interval, the BA is able to support the expression of avoidance even in the absence of LA inputs. Both LA and BA require projections from the auditory cortex in order to emit conditioned defensive behaviors. Therefore, we propose that LA is essential for the formation of the CS-US association but, over time, the CS-US memory that drives avoidance depends on a cortical-basal amygdala circuit which operates independently from a cortical-lateral and central amygdala circuit that is essential for freezing.

The data that LA lesions performed 15–20 mins after training impaired the formation of memories, irrespective of the fear index analyzed, is in line with previous studies showing that LA blockade performed before or shortly after training impaired the subsequent expression of freezing^34,35^ and avoidance^5,7,8^. Overall, these data support the idea that LA is essential for the formation of fearful memories. Conversely, the fact that LA lesions performed several weeks after training did not affect the expression of all types of defensive reactions sheds a new light on the participation of LA to long-term fearful memories. Previous studies have addressed the involvement of LA (or, more frequently, LA + BA) in the long-term expression of CS-US association. All these studies showed a permanent impairment in the freezing and conditioned startle responses to the CS^19–21^, and therefore come to the conclusion that this nucleus is a key substrate supporting long-term memory storage of fearful memories^22^. This view was further supported by data showing that the retrieval of long-term memories enhances LA activity^17,18^ but see^15^. Our data confirmed that LA lesions impaired freezing to threatening CSs. However, through the simultaneous measurement of an alternative fear index in the same animals, our data showed that the involvement of the LA in the long-term expression of fearful memories is much more limited than previously hypothesized, suggesting that the LA is not an essential locus for all CS-US memories. Our results did not allow to discriminate whether during the expression of long-term memories the LA serves only for the regulation of the freezing response or, alternatively, whether it also maintains memory information essential to drive the freezing response, as previously claimed^15,17–20^, but see^21^. In the latter case, our results suggested that within the amygdala there may be multiple and independent memory representations of the CS-US association.

The other major finding coming from our study is that the BA may operate independently from LA inputs during the expression of long-term memories, in order to bias the choice of action away from the source of the threat. This finding does not support the idea that LA and BA form an anatomically and functionally unitary complex (the basolateral complex, BLA)^4^ nor that LA is the main pathway carrying sensory information about the CS to the amygdala^1^. With respect to previous studies^5,6,36^ our data strongly suggest that, within the amygdala, there are at least two parallel input pathways, the LA and the BA. Two dissociable circuits originate from these two nuclei, one relying on the LA-to-CeA pathway which serves to express freezing to threatening stimuli, and the other relying solely on the BA (not on LA or CeA), which drives the actions of the subject away from the source of the threatening stimulus. These two pathways may support memory expression independently.

On the other hand, it remains unclear the role of the BA in the formation of recent fearful memories. This structure may be involved in the memory formation process as the LA nucleus. In this case, a lesion would impair the expression of both freezing and avoidance behavior. Alternatively, its role may be specific for the expression of avoidance behavior. Notably, in our previous work^14^, where animals could display only freezing response, BA was indeed activated after recent but not remote fearful memory expression, maybe suggesting its involvement in fear memory formation. Future studies will better address this issue.

**Figure 6:**
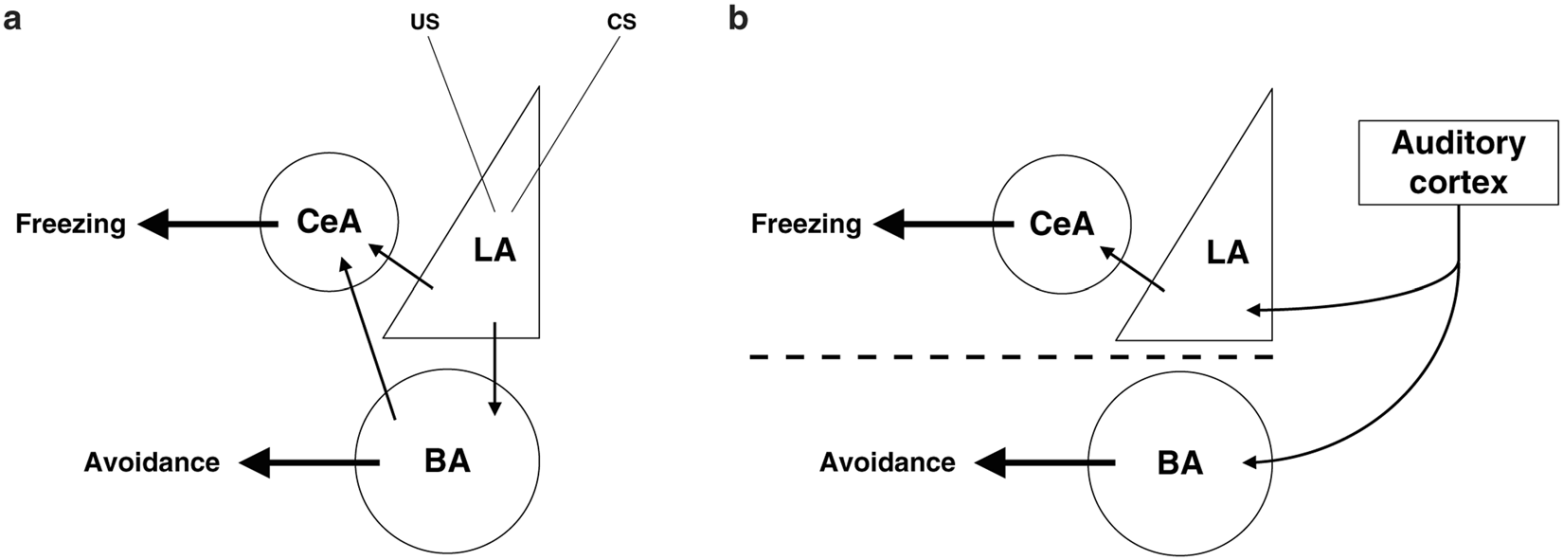
Two models of information processing in the amygdala. (**a**) The traditional model of information flow within the amygdala proposes that during the formation of fearful memories the LA is the input area for both conditioned (CS) and unconditioned (US) stimulus information. From this site, information is conveyed either directly or indirectly (through the BA) to the CeA (Adapted from Amorapanth *et al*., 2000). (**b**) The new model detailing the proposed neural circuits engaged within the amygdala during the long-term auditory fearful memory expression. In this model, the amygdala receives two independent and parallel inputs. One arrives at LA and through the CeA it supports freezing behavior in response to the aversive CS. The other input arrives at BA and drives the ability of rats to bias their actions away from the CS. The latter circuit operates independent from LA and CeA. In addition, we propose that essential information regarding auditory fearful memories arrives at either LA or BA from the auditory cortex (AC).

Overall, our findings lead to the idea that the flow of information that has been proposed to occur serially from LA to BA to CeA during the formation of a fearful memory trace (Fig. 6a), cannot be applied to the long-term expression of fearful memories in favor of a new model in which two parallel circuits operate independently and mediate distinct defensive responses (Fig. 6b).

Our study also revealed that both LA and BA require direct inputs from the auditory cortex, and particularly the Te2 area, in order to produce defensive responses. This data is in line with previous findings that during the retrieval of long-term fearful memories the activity of Te2 and LA + BA nuclei is highly synchronized in the theta frequency range, and a preponderant Te2-to-BLA directionality characterizes this dialogue^32^. In addition, here we showed that the auditory cortex may be a crucial site for the maintenance of fearful memories regardless of whether or not freezing or avoidance is used as a measure. As LA is necessary for the formation of all fearful memories, it may be proposed that upon memory encoding this nucleus enables the formation of new memory traces at the level of the auditory cortex. Accordingly, previous studies showed that during the formation of a memory trace LA promotes memory-related processes in the auditory cortex^37–41^. Subsequently, during memory retrieval, these cortical memory traces may drive the LA and BA activity in order to express conditioned defensive responses.

The existence of two parallel and independent pathways within the amygdala has important implications for the study of anxiety disorders. Understanding the role of these two independent circuits is important for the treatment of traumatic memories, since the treatment often occurs long after the initial trauma^2^. In addition, studies addressing the possible extinction of traumatic memories should take into consideration these two independent and parallel pathways. Moreover, different exacerbated fear responses, such as avoidance behavior in obsessive-compulsive disorders or immobility during panic attacks, may rely on different circuits in the amygdala.

## Methods

### Subjects

Male Wistar rats (age: 65–75 days, weight: 230–360 g) were used. All the animals were housed in plastic cages with food and water available ad libitum, under a 12 h light/dark cycle (lights on at 7:00 A.M.) at a constant temperature of 22 ± 1 °C. All the experiments were conducted in accordance with the European Communities Council Directive 2010/63/EU and approved by the Italian Ministry of Health (Authorization No 265/2011) and by the local Bioethical Committee of the University of Turin.

### Fear Conditioning

One auditory stimulus (pure tone, 8 s, 80 dB, 3.000 Hz, 22 s inter-trial interval) was administered repeatedly for seven times as conditioned stimulus (CS), each time co-terminating with a painful stimulation (unconditioned stimulus, US) consisting of an electric foot shock (1 s, 0.7 mA). We employed seven trials because previous studies using this protocol^14,30,42^ reported a strong fear memory (freezing levels ≥80%) lasting for 4–5 weeks. Tones were delivered by a loudspeaker located 20 cm above the grid floor. Rats were left in the chamber for 1 additional min, and then they returned to the home cage.

### Fearful Memory Test

Long-term aversive memories were tested at five weeks after the conditioning session. Animals were handled for four days before memory retention. Both habituation and retention occurred in a completely different apparatus, which was located in a separate experimental room in order to avoid conditioned fear responses to contextual cues^14^.

#### Apparatus

The testing chamber was a long cage made of 2 cm thick fir wood (184 cm length x 40 cm height x 34 cm width, opened on the superior side), internally covered with wood texture laminated paper. The cage was divided in six adjacent zones (each 30 × 30 cm) by a 2 cm wide strip of green adhesive tape applied to the ground, for a total of 180 × 30 cm of accessible space (Fig. 1a). On the external side of the short wall in zone 6, a loudspeaker and a light bulb were respectively fixed 6 cm and 13 cm above the ground, horizontally centered. Both the loudspeaker and the light bulb were pointed into the accessible space through two holes and were not visible from inside the cage. An exhaust fan, which eliminated odorized air from the enclosure, provided background noise of 60 dB.

#### Procedure

Animals underwent a four-days habituation protocol and a memory retention test on the fifth day. On the first day rats were delicately placed into the long cage (zone 2) with the snout oriented towards the light and auditory sources (zone 6) and left undisturbed for 8 min, then they returned to their home cages. The same procedure was repeated on the second day. In the third day, in order to increase the exploration of the zone 6 (i.e., the zone where the CS will be administered), a child’s toy consisting in a light blue building block (6,5 cm length x 5,5 cm height x 3,5 cm width) was added into the cage (fixed on the short wall) in zone 6, and animals were left free to explore it for 5 min. The same procedure was repeated on the fourth day. On the fifth day, the control stimulus was removed, and memory retention was assessed through the presentation of the CS. As in the previous days, rats were placed into the apparatus (zone 2). To facilitate the identification of the source of the CS, a light stimulus was delivered from the bulb located above the loudspeaker. When rats were in zone 2 with the snout oriented towards the light and auditory sources (zone 6), the light turned on and, 2 s later, the first CS was delivered. All the successive CSs were simultaneously presented with the light, for a total amount of 6 tones-lights of 8 s. CSs were administered in a manner identical to that employed during the conditioning session (duration, intensity and inter-trial intervals). The innate threat stimulus consisted in an eagle-shaped child toy, which was presented 1 m above the cage for 3 times of 8 s each, with an inter-trial interval of 22 s. The rat behavior was recorded by means of a digital video camera, fixed to the ceiling of the room and pointed perpendicularly to the apparatus.

### Avoidance and Freezing Analysis

Behavioral patterns used as fear indices were avoidance and freezing responses. *Avoidance* was defined as the difference between the time that rats spent in zone 1 and in zone 6, measured during the 180-s fear memory retention test. The time that rats spent in each zone of the apparatus was analyzed using Smart v. 3.0.04 (Panlab Harvard Apparatus). *Freezing* response was defined as the complete absence of somatic mobility, except for respiratory movements. Freezing was measured by means of a stopwatch and counted as the overall time of immobility within the overall accessible space (zones 1–6) of the cage. A further employed index was the *travelled distance*. The measurement was performed using Smart v. 3.0.04. The following formula was used: *Travelled distance* = [(*d* * 180)/*l*]/100, where *d* is the raw travelled distance (cm) calculated by the software, 180 is the constant real length (cm) of the cage, and *l* is the length (cm) of the cage estimated by the software. All the behavioral analyses were performed by two persons who did not know to which experimental group each animal belonged (E. M. and A. R.).

### Surgery

#### Irreversible excitotoxic lesions

Rats were mounted in a stereotaxic apparatus, an incision of the skull was made, and small burr holes were drilled to allow the penetration of a 28 gauge infusion needle. A 10 µl Hamilton syringe mounted on an infusion pump was used to deliver infusions at a rate of 0.1 µl/min. Neuronal cell loss was induced by injecting the ibotenic acid^43^ or the N-methyl-D-aspartic acid (NMDA) bilaterally at the coordinates taken from Paxinos and Watson atlas^44^. The needle was left in place for additional 6 mins in the case of CeA, LA and Te2 lesions and of 10 mins in the case of BA lesions. Naive and conditioned sham rats underwent an identical procedure, except that no infusions were made. Because no differences were detected among sham-operated animals in CeA, LA (15–20 mins after as well as four weeks after training), BA or Te2 areas, they were collected altogether. For this reason, the same naive and conditioned animals were kept constant throughout all the experiments. Following the surgical procedures, the rats were kept warm and under observation until recovery from anesthesia. All behavioral procedures were made one week after surgery to allow for recovery.

#### Amygdala nuclei lesions

*Central amygdala lesions* were made by administering ibotenic acid (10 mg/ml, dissolved in sodium phosphate buffer, pH 7.4) at two points with the following coordinates: 1.9 mm posterior (AP) to bregma, ± 4.0 mm lateral (L) and 8.4 mm ventral (V), total volume 0.20 µl; 2.5 mm posterior to bregma, ± 4.3 mm lateral and 8.2 mm ventral, total volume 0.22 µl. *Lateral amygdala lesions* were made by administering NMDA (18 mg/ml, dissolved in physiological saline) at two points with the following coordinates: 2.3 mm posterior (AP) to bregma, ±5.4 mm lateral (L) and 7.9 mm ventral (V), total volume 0.18 µl; 3.3 mm posterior to bregma, ±5.4 mm lateral and 8.0 mm ventral, total volume 0.18 µl. *Basal amygdala lesions* were made by administering NMDA (16 mg/ml) at two points with the following coordinates: 2.28 mm posterior (AP) to bregma, ±4.7 mm lateral (L) and 9.65 mm ventral (V), total volume 0.15 µl; 3.3 mm posterior to bregma, ±4.7 mm lateral and 9.65 mm ventral, total volume 0.16 µl.

#### Secondary auditory cortex lesions

Secondary auditory cortex Te2 was lesioned by injecting NMDA (18 µg/µl) at the coordinates: AP = 5.8, L = ± 6.5–6.7, V = 6.0, 0.22 µl; AP = 6.8, L = ± 6.5–6.7, V = 6.0, 0.22 µl.

### Histology

The extension of the damaged areas in the case of excitotoxic lesions was histologically verified at the end of the experiments with NeuN staining analysis. Rats were intracardially perfused with 4% PFA. The brain was incubated in 4% PFA overnight at 4 °C, transferred to 30% sucrose and finally sectioned at 30 μm on a cryostat. Sections were placed in NeuN antibody (1:2000) at room temperature (RT) for 24 h, transferred to biotinylated anti-mouse antibody IgG (1:1000) at RT for 1 h and finally placed in a solution of ABC on a shaker for 2 h. Sections were rinsed in Tris-HCl and incubated in DAB. The DAB was developed by the addition of 0.015% H_2_0_2_, and the development stopped by washing with Tris-HCl. Verification of lesions was made observing NeuN-stained tissue under a microscope magnified at 2x and 4x. The lack of staining was used as an indication of neuronal loss due to lesions. The extensions of the lesions were analyzed by two persons who did not know to which experimental group each animal belonged (L. M. and B. S.).

### Virus-mediated gene expression

The adeno-associated viruses (AAV, serotype 5) were obtained from the University of North Carolina Vector Core (Chapel Hill, NC, USA). Viral titer was 4.7 × 10^12^ vg/ml for AAV5:CaMKIIα::eNpHR3.0-mCherry. The use of CaMKII promoter enables transgene expression favoring pyramidal neurons. Viruses were housed in an −80 °C freezer. Viral infusions targeting the Te2 cortex were performed at the two stereotaxic coordinates employed for the excitotoxic lesions at the volumes of 0.5 μl. The viruses were injected at a rate of 0.1 μl/min, and the needle was left in place for additional 10 min. These injections produced strong opsin expression in the Te2 cortex. Fibers from the Te2 cortex were found to innervate either the lateral and basal amygdala, in keeping with prior anatomy studies^25–27^. The optic fibers (200/230 μm core; 10 mm length, Plexon, Texas, USA) were implanted bilaterally in LA + BA (AP = −2.8, L = ± 5.4, V = 8.2 mm from bregma).

### Illumination

Optogenetic inhibition of Te2 projections to LA + BA was obtained by using the PlexBright Optogenetic Stimulation System (Plexon, Texas, USA). Orange light (620 nm) was generated by Compact LED Modules (Plexon, Texas, USA). Light was passed through an optical fiber (2.40 m lenght) to reach the brain. The power density estimated at the tip of the optic fiber was of 3 mW for illumination of projection sites. Orange light was initiated 4 s prior to CS/innate threat stimulus onset, persisted throughout the 8 s CS/innate threat stimulus, and was stopped 4 s after the CS/innate threat stimulus offset. Rats were familiarized with the patchcord for 4 days before starting each behavioral session.

### mCherry immunohistochemistry

Upon completion of experiments, rats were deeply anaesthetized and perfused intracardially with 4% PFA in order to examine the diffusion of the virus. The brains were dissected, stored overnight at 4 °C, and finally transferred to 30% sucrose. Coronal sections (50 μm) were cut on a cryostat and collected in PBS. Free-floating sections were incubated in a blocking solution (4% bovine serum albumin (BSA), 10% normal goat serum and 0.5% Triton X-100) for 1 h at RT. Then, they were incubated in primary monoclonal mouse antibody to mCherry (1: 500 dilution, Abcam, ab125096) in the blocking solution overnight at 4°. Subsequently, sections were washed with PBS and incubated for 1 h at RT with secondary fluorescent labels AlexaFluor-568-labeled anti-mouse antibody (1:400 dilution, Lifetechnologies, A11036) diluted in PBS for 1 h on a shaker at room temperature. Sections were washed in PBS, mounted with mounting media containing DAPI (Vector, H1200) and coverslipped.

### Confocal microscope imaging

The presence of mCherry labeling was examined by using a Leica SP5 confocal microscope: two laser were used (488 and 570 nm), each corresponding to the peak emission spectrum for DAPI (Nissl stain for cell nuclei) and CY3 (mCherry), respectively. Representative photomicrographs of Te2 and LA + BA (Fig. 5g) are mosaic images (each single was acquired by using a 20 × objective).

In order to visualize Te2-LA and Te2-BA terminals, we acquired Z-stack images in both areas by using a 63 × objective. Thus, LA and BA images showed in Fig. 5g are maximum intensity projections of 5 optical sections spaced 1 μm apart.

### Statistical analysis

Since all data passed the Levene’s test for equality of variances, parametric statistics were employed through all the experiments. In order to test the main effect of *zones* (and specifically zone 1 and 6) and the simple main effects of *groups* (naive, conditioned and lesioned or mCherry control and eNpHR-mCherry), a mixed-design ANOVA with *group* as a between-subjects variable and *zone* as a within-subjects variable was performed. Pairwise multiple comparisons for the simple main effects of groups were implemented using Fisher’s Least Significant Difference (LSD) tests, corrected with Bonferroni adjustments. In order to test the differences between groups on the avoidance response, and the differences between groups on the freezing response, a one-way ANOVA (or a Student’s *t* test, in the case of two groups) was performed. *Post hoc* analysis was implemented using Bonferroni-corrected *t* tests. All statistical analyses were performed using SPSS Statistics 22 (IBM).

### Data availability

The datasets generated during and analysed during the current study are available from the corresponding author on reasonable request.

## Electronic supplementary material

Supplementary information
