# Supplementary material for: Lateral and Basal Amygdala Account for Opposite Behavioral Responses during the Long-Term Expression of Fearful Memories
Source: Sci Rep. 2018 Jan 11;8:518. doi: 10.1038/s41598-017-19074-3 (PMC5765149; doi:10.1038/s41598-017-19074-3)
Supplement: Supplementary file 1 — Supplementary information [file 41598_2017_19074_MOESM1_ESM.pdf]

Lateral and Basal Amygdala Account for Opposite Behavioral Responses during the Long-Term Expression of Fearful Memories

Authors: Eugenio Manassero, Annamaria Renna, Luisella Milano and Benedetto Sacchetti

Supplementary Information

Supplementary Figures and Figure Legends

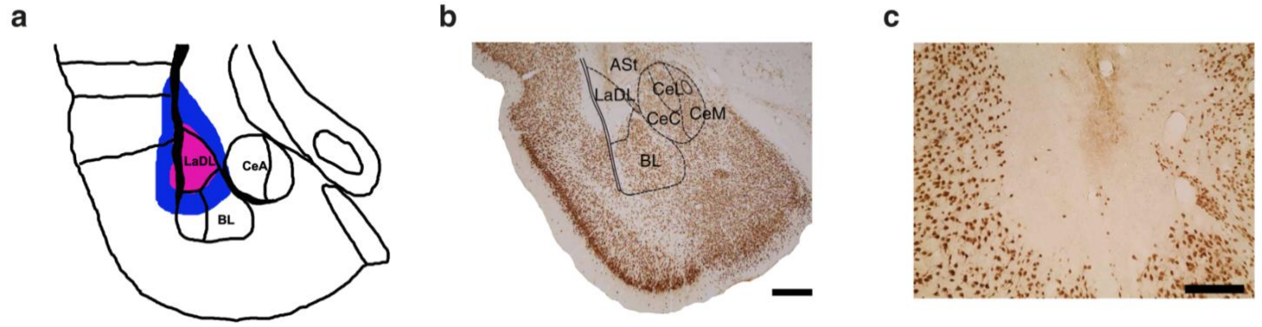

Supplementary Figure S1. Lesions of the lateral nucleus of the amygdala (LA). (**a**) Exemplification of the smallest (red-violet) and the widest (blue) excitotoxic lesion of the LA. An overall number of 36 rats underwent lesion in LA. Among these, 24 animals were excluded from the analysis due to different reasons. In particular, 6 of them showed an incomplete bilateral damage, while for 15 of them the lesioned area affected the BA and/or the CeA. Moreover, 3 rats showed a lesion not in LA but only above the LA, i.e. to the caudate putamen (striatum, CPu); notably, these 3 subjects did not show an amnesic behavior (data not shown). The 12 animals that were part of the sample were specifically damaged in the LA, encompassing the dorsolateral (LaDL), ventromedial (LaVM) and ventrolateral (LaVL) part of the lateral nucleus. In some cases, the damage marginally extended to the amygdalostriatal transition (ASt), to the adjacent perirhinal cortex (PRh) and to a very restricted portion of BA nucleus. The section diagram was drawn on the basis of our NeuN-stained section. (**b**) Coronal sections through LA lesions stained with NeuN antibody. Scale bar, 500 µm. (**c**) The N-methyl-D-aspartic acid (NMDA) injection determined neuronal death within the LA. Scale bar, 200 µm. ASt, amygdalostriatal transition; BL, basolateral amygdaloid nucleus; CeA, central amygdaloid nucleus; CeC, central amygdaloid nucleus, capsular division; CeL, central amygdaloid nucleus, lateral division; CeM, central amygdaloid nucleus, medial division; LaDL, lateral amygdaloid nucleus, dorsolateral part.

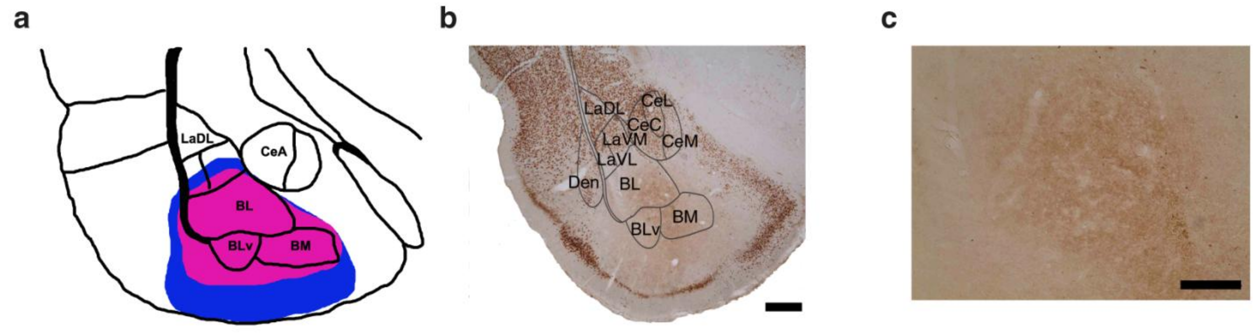

Supplementary Figure S2. Lesions of the basal nucleus of the amygdala (BA). (**a**) Exemplification of the smallest (red-violet) and the widest (blue) excitotoxic lesion of the BA. From 35 animals that were damaged to the BA, 16 ones were excluded because the lesion extended to the central (CeA) and/or the lateral (LA) amygdaloid nuclei. Other animals (*n* = 6) were not included in the analysis because of an incomplete bilateral damage. The rats that were included in the sample (*n* = 13) were focally damaged in the BA, encompassing the basolateral (BL), ventral basolateral (BLv) and basomedial (BM) subnuclei. In some cases, the damage partially extended to the adjacent entorhinal and piriform cortex. The section diagram was drawn on the basis of our NeuN-stained section. (**b**) Representative photomicrograph of NeuN-staining of the BA lesion. Scale bar, 500 µm (**c**) NMDA administration determined neuronal death within the BA. Scale bar, 200 µm. BL, basolateral amygdaloid nucleus; BLv, basolateral amygdaloid nucleus, ventral part; BM, basomedial amygdaloid nucleus; CeA, central amygdaloid nucleus; CeC, central amygdaloid nucleus, capsular division; CeL, central amygdaloid nucleus, lateral division; CeM, central amygdaloid nucleus, medial division; Den, dorsal endopiriform nucleus; LaDL, lateral amygdaloid nucleus, dorsolateral part; LaVL, lateral amygdaloid nucleus, ventrolateral part; LaVM, lateral amygdaloid nucleus, ventromedial part.

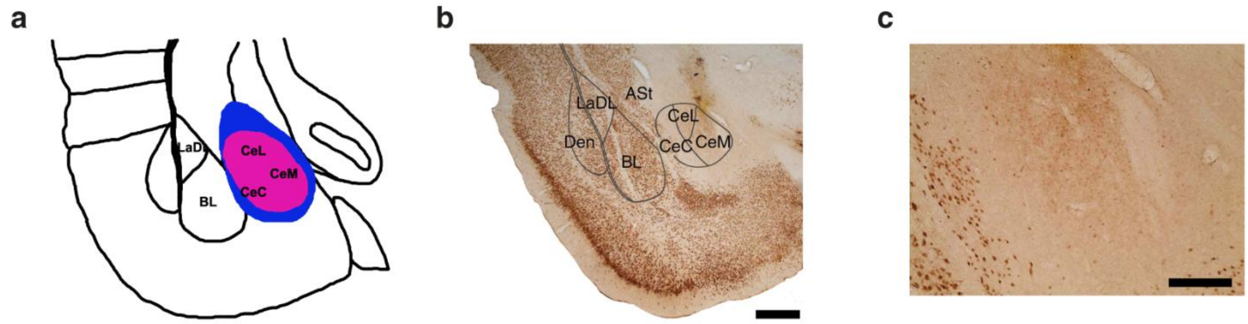

Supplementary Figure S3. Lesions of the central nucleus of the amygdala (CeA). (**a**) Exemplification of the smallest (red-violet) and the widest (blue) excitotoxic lesion of the CeA. From 27 animals that were lesioned, 18 rats were excluded from the sample. More specifically, 11 animals were not included in the analysis because the damaged area partially extended to the adjacent lateral (LA) and/or basal (BA) amygdaloid nuclei. The remaining rats (n = 7) were excluded due to an incomplete bilateral lesion. The included animals (n = 9) were focally lesioned in the CeA, encompassing the medial (CeM), capsular (CeC) and lateral (CeL) divisions of the central amygdala. Only in some cases there was a tiny damage to the globus pallidus (GP). The section diagram was drawn on the basis of our NeuN-stained section. (**b**) Coronal photomicrograph of NeuN staining of CeA damage. Scale bar, 500 µm. (**c**) The ibotenic acid injection induced neuronal loss within the CeA. Scale bar, 200 µm. ASt, amygdalostriatal transition; BL, basolateral amygdaloid nucleus; CeC, central amygdaloid nucleus, capsular division; CeL, central amygdaloid nucleus, lateral division; CeM, central amygdaloid nucleus, medial division; Den, dorsal endopiriform nucleus; LaDL, lateral amygdaloid nucleus, dorsolateral part.

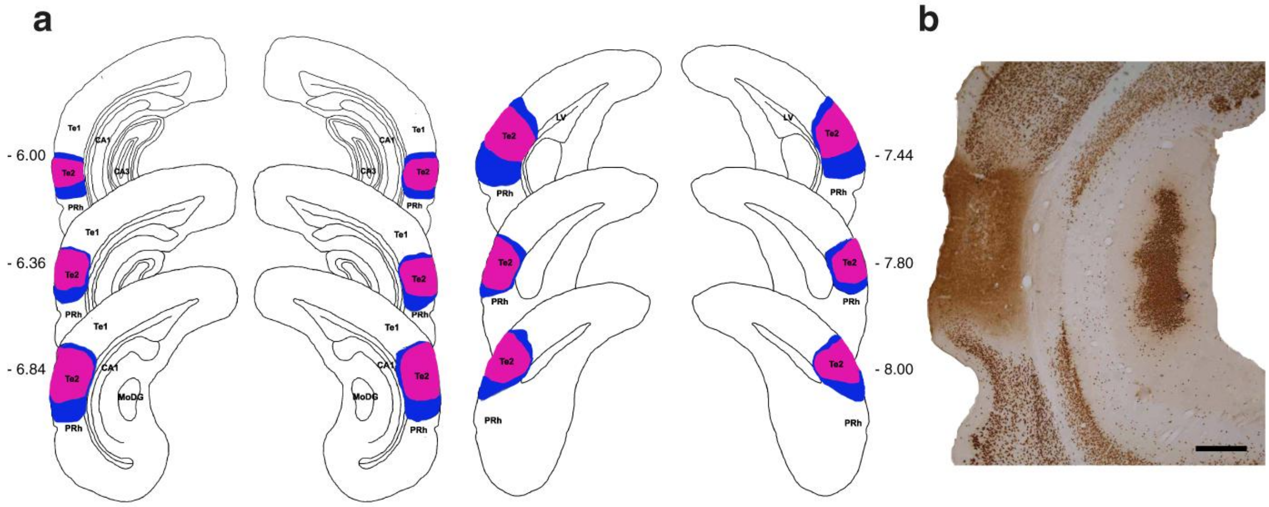

Supplementary Figure S4. Secondary auditory cortex (Te2) lesions. (**a**) Narrowest (red-violet) and largest (blue) damage of the secondary auditory cortex (Te2). An overall amount of 16 rats underwent Te2 lesion. From these animals, 7 were excluded because the bilateral damage was not extended to the entire portion of the cortex. The animals that were included in the analysis (n = 9) showed a focal and bilateral cell loss in the Te2 area. In some cases, the lesion extended to the occipital cortex above the Te2 area and to the posterior perirhinal cortex. Negative numbers indicate posterior distance from bregma. The serial section diagram was drawn on the basis of our NeuN-stained sections. (**b**) Coronal photomicrograph of a representative Te2 lesion, stained with NeuN antibody. Scale bar, 500 μm. CA1, field CA1 of the hippocampus; CA3, field CA3 of the hippocampus; MoDG, molecular layer dentate gyrus; PRh, perirhinal cortex; Te1, primary auditory cortex; Te2, secondary auditory cortex.

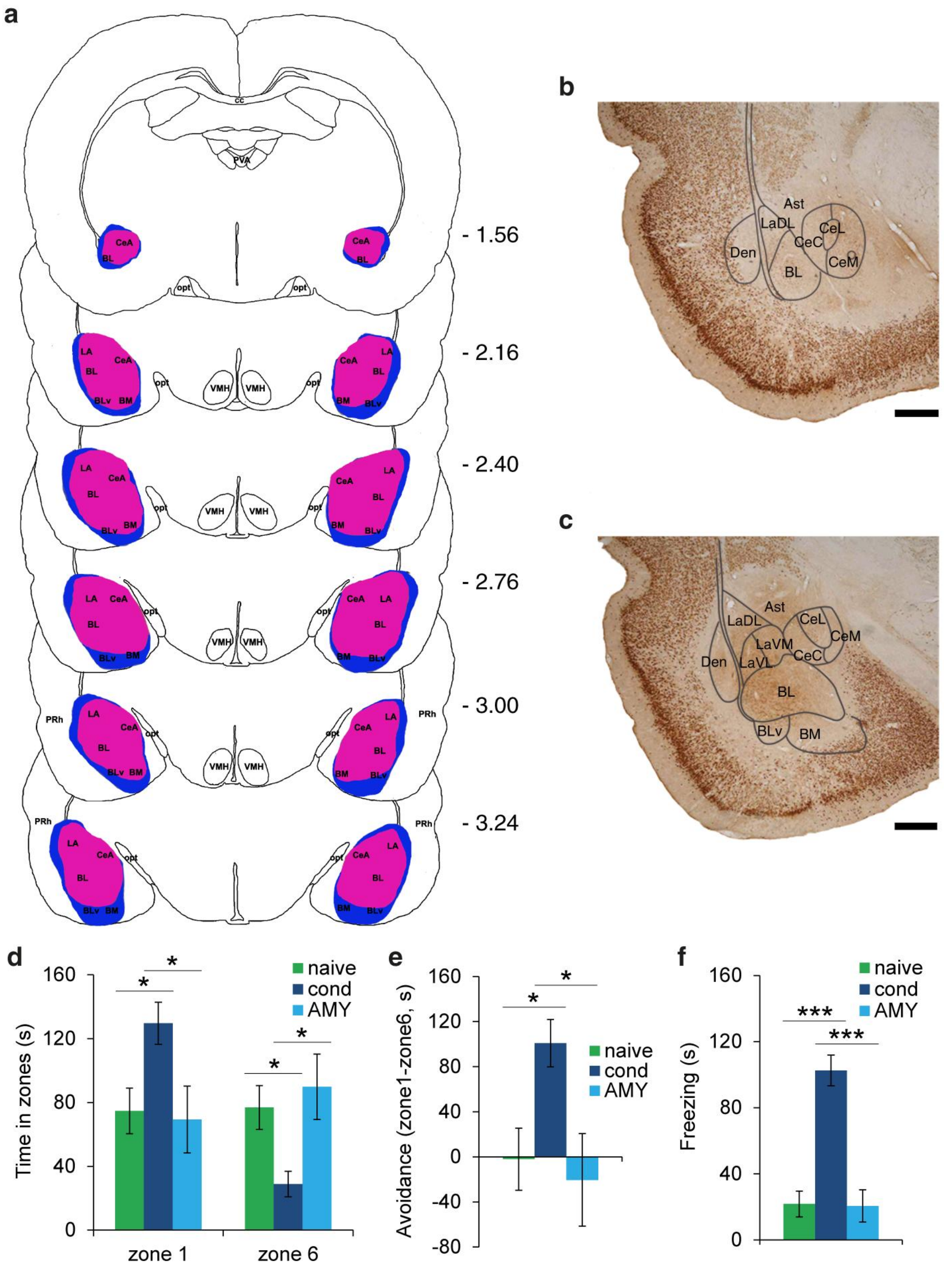
a
cc
PVA
CeA
BL
opt
- 1.56
LA
BLv
BM
VMH
- 2.16
- 2.40
- 2.76
PRh
- 3.00
- 3.24
b
Ast
LaDL
CeL
CeC
Den
BL
CeM
c
LaVM
LaVL
d
Time in zones (s)
naive
cond
AMY
zone 1
zone 6
e
Avoidance (zone1-zone6, s)
f
Freezing (s)

Supplementary Figure S5. Irreversible lesions of the entire amygdala and long-term fearful memories retention. (**a**) Extent of the narrowest (red-violet) and the largest (blue) damage of the entire amygdala (n = 8). From 12 animals that were lesioned, 4 rats were not included in the analysis because of an incomplete bilateral lesion. The included animals (n = 8) were lesioned in the CeA, LA and BA. In some cases, there was a damage to the adjacent perirhinal, piriform and entorhinal cortex. The serial section diagram was drawn on the basis of our NeuN-stained sections. (**b, c**) Representative photomicrographs of the amygdala lesions (NeuN-staining). Scale bars, 500 µm. (**d**) Time spent in zone 1 and zone 6 during the memory test, by naive (n = 13), conditioned (n = 15) and AMY-lesioned rats (n = 8). A mixed-design ANOVA yielded significant main effects of the group in zone 1 ($F_{(2,33)}$ = 5.07, P = 0.012) and in zone 6 ($F_{(2,33)}$ = 6.32, P = 0.005). Pairwise Bonferroni-corrected comparisons indicated that the time spent in zones 1 and 6 was similar between naive and AMY-lesioned animals (P > 0.05) but differed between conditioned and AMY-lesioned animals in zone 1 (P = 0.042) and zone 6 (P = 0.011). (**e**) Avoidance behavior ($F_{(2,33)}$ = 5.78, P = 0.007) was weaker in animals lesioned in the entire amygdala than in conditioned subjects (P = 0.021), while it was not different from naive ones (P > 0.05). (**f**) Freezing responses in AMY-lesioned rats ($F_{(2,33)}$ = 29.1, P < 0.001) were similar to naive animals (P > 0.05) and different to conditioned ones (P < 0.001). * P < 0.05; ** P < 0.01; *** P < 0.005. All values are reported as mean ± SEM. ASt, amygdalostriatal transition; BL, basolateral amygdaloid nucleus; BLv, basolateral amygdaloid nucleus, ventral part; BM, basomedial amygdaloid nucleus; cc, corpus callosum; CeA, central amygdaloid nucleus; CeC, central amygdaloid nucleus, capsular division; CeL, central amygdaloid nucleus, lateral division; CeM, central amygdaloid nucleus, medial division; Den, dorsal endopiriform nucleus; LA, lateral amygdaloid nucleus; LaDL, lateral amygdaloid nucleus, dorsolateral part; LaVL, lateral amygdaloid nucleus, ventrolateral part; LaVM, lateral amygdaloid nucleus, ventromedial part; opt, optic tract; PRh, perirhinal cortex; PVA, paraventricular thalamic nucleus, anterior part; VMH, ventromedial hypothalamic nucleus.
